# Atypical Hemolytic Uremic Syndrome Secondary to Pancreatitis: A Case Report

**DOI:** 10.7759/cureus.35434

**Published:** 2023-02-24

**Authors:** Tsubasa Kajiyama, Masahumi Fukuda, Yuuichirou Rikitake, Osamu Takasu

**Affiliations:** 1 Emergency Medicine, Advanced Emergency and Critical Care Center, Kurume University Hospital, Kurume, JPN; 2 Gastroenterology, Advanced Emergency and Critical Care Center, Kurume University School of Medicine, Kurume, JPN; 3 Intensive Care Unit, Advanced Emergency and Critical Care Center, Kurume University School of Medicine, Kurume, JPN

**Keywords:** pancreatitis, thrombotic microangiopathy (tma), hus, ahus, atypical hemolytic uremic syndrome

## Abstract

This is a report of an extremely rare case of an atypical hemolytic uremic syndrome (aHUS) that appears to have been triggered by acute pancreatitis. A 68-year-old man was examined at a medical institution because of sudden lower abdominal pain. The patient was diagnosed with acute pancreatitis on computed tomography. Hemoglobinuria and laboratory findings indicative of intravascular hemolysis were noted. Biochemical analysis revealed normal results for von Willebrand factor activity, antiplatelet antibodies, and ADAMTS13 (a disintegrin and metalloproteinase with thrombospondin type 1 motif, member 13), and stool culture was negative for Shiga-toxin-producing *Escherichia coli*, leading to the diagnosis of aHUS. Treatment for acute pancreatitis resulted in improvement in the laboratory findings, and the patient’s progress was monitored without treatment intervention for aHUS. On day 2 of hospitalization, the abdominal symptoms and hemoglobinuria resolved without any subsequent recurrence. In the absence of any complications, the patient was transferred back to the initial hospital on day 26 of hospitalization. When hemolytic anemia or thrombocytopenia of unknown etiology is observed, aHUS should be suspected, and clinicians should be aware that acute pancreatitis may be a potential cause of aHUS.

## Introduction

Thrombotic microangiopathy (TMA) is a syndrome caused by the formation of platelet thrombi in small blood vessels and is known to cause thrombocytopenia, microangiopathic hemolytic anemia, and organ injury [1]. The most common types of TMA are thrombotic thrombocytopenic purpura (TTP) and hemolytic uremic syndrome (HUS), also referred to as STEC-HUS, which are caused by Shiga-toxin-producing *Escherichia coli* (STEC) infections.

Meanwhile, HUS that is not caused by STEC infection is referred to as atypical HUS (aHUS), the reported causes of which include complement dysregulation in addition to a variety of factors such as drugs, malignant tumors, and pregnancy [2]. Infections and pregnancy are frequent triggering events [3], among which infections caused by *Streptococcus pneumoniae* account for 40% of aHUS episodes [4].

In addition to the fact that aHUS is an extremely rare disease, with a prevalence of 4.96 per million total population [5], cases of aHUS triggered by acute pancreatitis are extremely rare in the literature, having been reported only by Barish et al. [6]. This is a case report of an aHUS that appears to have been triggered by acute pancreatitis. The patient provided written consent for the presentation of this case in this article.

## Case presentation

The patient was a 68-year-old man with no family history of coagulopathy and no history of heavy alcohol consumption. He was on oral treatment, as prescribed by his primary physician, for diabetes mellitus (vildagliptin 100 mg/day and metformin 500 mg/day), hypertension (telmisartan 40 mg/day), and dyslipidemia (bezafibrate 800 mg/day). There was no change in the oral medication within three months.

Lower abdominal pain suddenly developed, and the patient decided to self-monitor his progress for about half a day. He experienced nausea and vomiting with no improvement; therefore, he was examined on the same day at the initial hospital. The patient was diagnosed with severe acute pancreatitis on abdominal computed tomography (CT) at the hospital and was transferred to our facility for treatment. Upon arrival at the emergency department (ED), the patient presented with a blood pressure of 162/101 mmHg, a pulse of 107 bpm, a respiratory rate of 34 bpm, a temperature of 38.1°C, tachycardia, and tachypnea with fever. No disturbance of consciousness was observed. Physical examination revealed jaundice in the orbital conjunctiva but no evidence of anemia in the palpebral conjunctiva. Tenderness in the middle lower abdomen and diminished bowel peristalsis were noted. There was no evidence of Cullen’s sign, Grey-Turner’s sign, or Fox’s sign. Physical findings of pancreatitis were limited.

**Figure 1:**
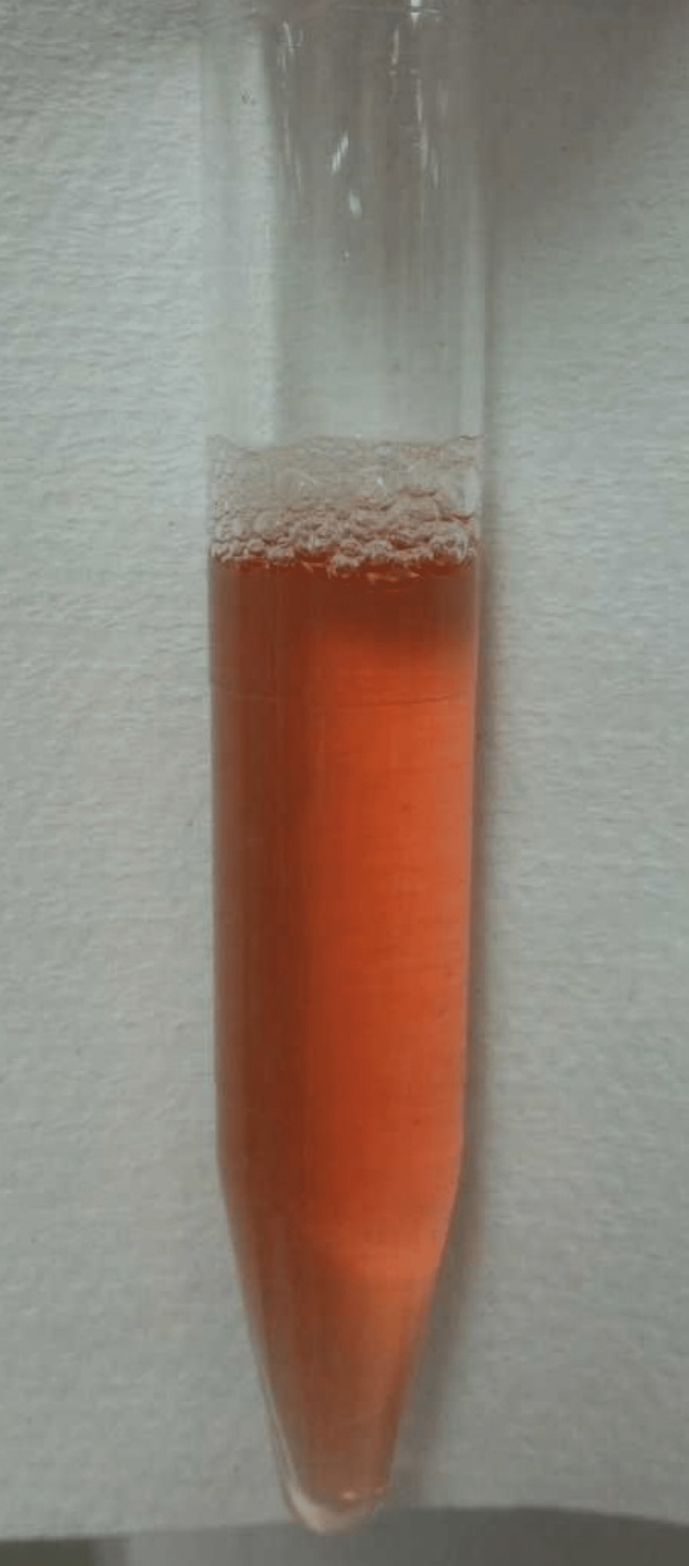
Macroscopic hemoglobinuria

Macroscopic hemoglobinuria (Figure 1) was confirmed upon arrival at the ED; however, when interviewed, the patient reported no obvious urine abnormalities before the onset of abdominal pain.

**Figure 2:**
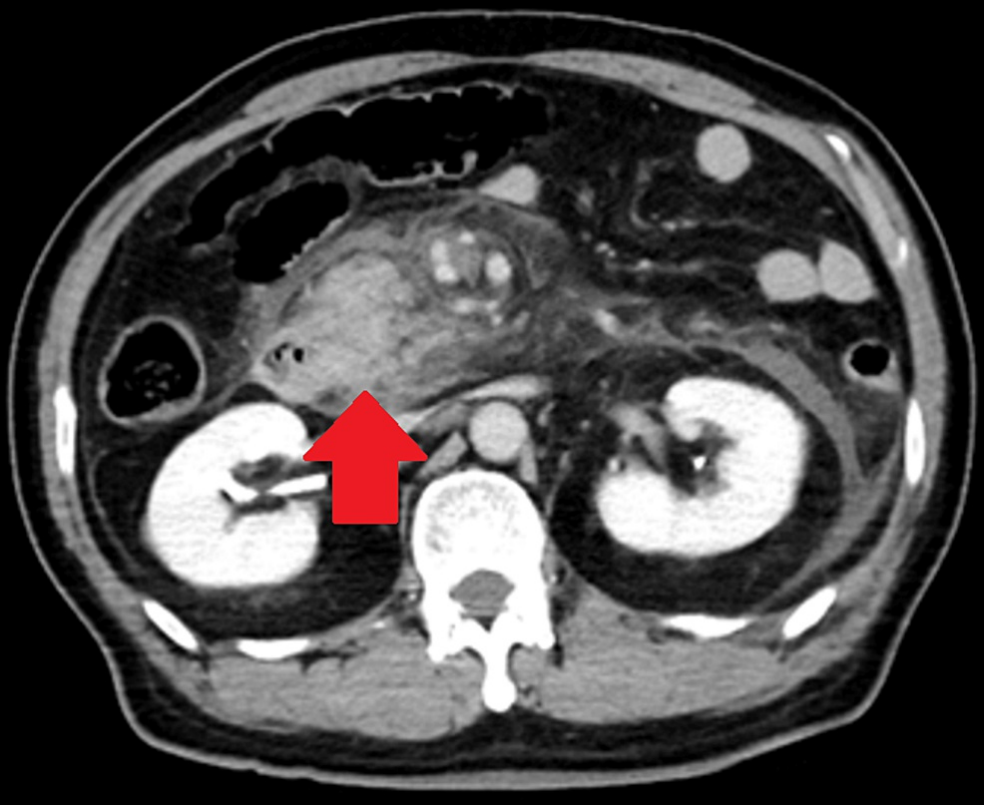
Contrast-enhanced abdominal CT indicating the inflammatory findings in the abdominal cavity (red arrows)

**Figure 3:**
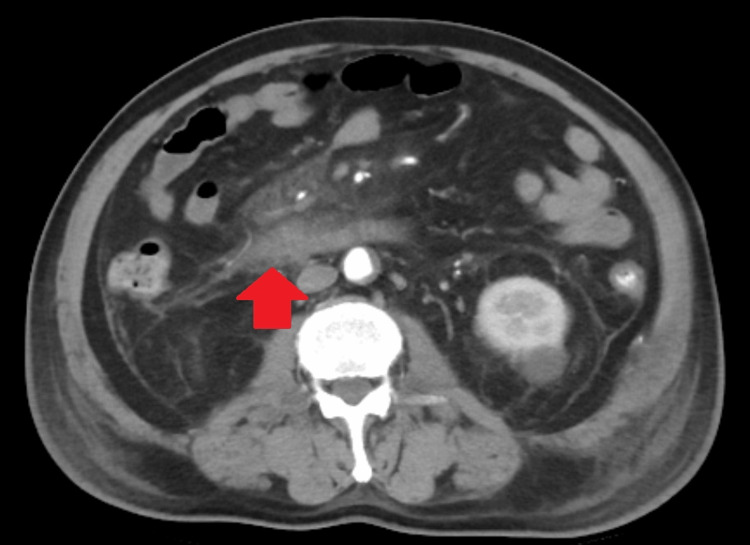
Contrast-enhanced abdominal CT indicating the spread of inflammation in the abdominal cavity (red arrows)

**Figure 4:**
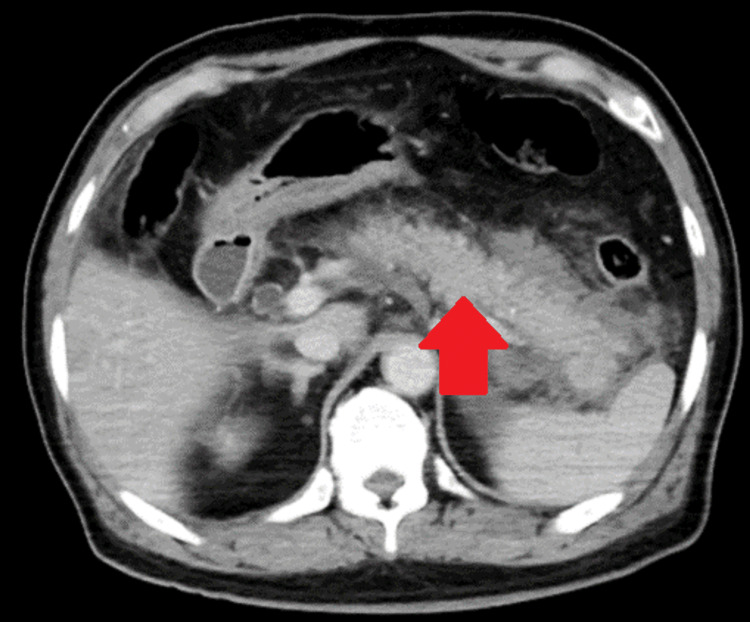
Contrast-enhanced abdominal CT indicating pancreatic parenchyma and the surrounding inflammation (red arrows)

Contrast-enhanced abdominal CT performed after arrival revealed no poorly visualized areas from the pancreatic head to the pancreatic tail, but extrahepatic adipose tissue inflammation had spread beyond the inferior pole of the kidney (Figures 2-4). There were no evident pancreatic tumors.

Table 1 presents biochemical findings on arrival. Elevated white blood cell count (18500/μL) and elevated C-reactive protein (33.23 mg/dL), and elevated pancreatic amylase (604 U/L) were consistent with pancreatitis. Antinuclear antibodies, immunoglobulin G, and triglycerides were analyzed to differentiate the cause of pancreatitis, but the results showed no abnormalities. Elevated creatinine and blood urea nitrogen levels also reflected the influence of chronic kidney disease. There was no evidence of anemia, but reticulocytes, bilirubin, and haptoglobin were evaluated because intravascular hemolysis was suspected due to elevated lactate dehydrogenase (LDH) (1519 U/L) and macroscopic hemoglobinuria. Intravascular hemolysis was indicated by elevated reticulocytes (70,300/μL) and total bilirubin (5.9 mg/dL), haptoglobin below the detection threshold, and the results of biochemical analysis.

**Table 1:** Laboratory data on admission WBC: White blood cell; RBC: Red blood cell; Hb: Hemoglobin; Ht: Hematocrit; Plt: Platelet; pH: Power of hydrogen; PT: Prothrombin time (percentage) activity; PT-INR: Prothrombin time and international normalized ratio; APTT: Activated partial thromboplastin time; Fbg: Fibrinogen; FDP: Fibrin degradation products; AT3: Antithrombin3; Na: Sodium; K: Potassium; Cl: Chloride; AST: Aspartate aminotransferase; ALT: Alanine aminotransferase; ALP: Alkaline phosphatase total; γ₋GTP: Gamma-glutamyl transpeptidase; Amy: Amylase; T. bil: Total bilirubin; D. bil: Direct bilirubin; BUN: Blood urea nitrogen; Cre: Creatinine; CRP: C-reactive protein; LDH: Lactate dehydrogenase.

Laboratory data		Reference range
WBC	18,500/μL	3,300-8,600/µL
RBC	4.46 × 10^6^/μL	4.35-4.92 × 10^6^/µL
Hb	13.8 g/dL	11.6-14.8 g/dL
Ht	38.7%	35.1%-44.4%
Plt	121 × 10^3^/μL	158-348 × 10^3^/µL
PT activity	83%	80%-120%
PT-INR	1.12	0.85-1.15
APTT	17.9 seconds	24.0-39.0 seconds
Fbg	17.9 seconds	24.0-39.0 seconds
D-dimer	700 mg/dL	200-400 mg/dL
FDP	8.2 µg/mL	1.0 ≤ µg/mL
AT3	28.8 µg/mL	5.0 ≤ µg/mL
CRP	33.23 mg/dL	≤0.14 mg/dL
Lactic acid	2.47 mmol/L	0.56-1.39 mmol/L
LDH	1519 IU/L	120-220 IU/L
Na	134 mEq/L	138-145 mEq/L
K	4.5 mEq/L	3.6-4.8 mEq/L
Cl	102 mEq/L	101-108 mEq/L
AST	130 IU/L	13-30 IU/L
ALT	397 IU/L	10-30 IU/L
ALP	524 U/L	106₋322 U/L
γ₋GTP	725 U/L	13₋64 U/L
Ca	7.6 mg/dL	8.8₋10.1 mg/dL
IgG	1364 mg/dL	861₋1747 mg/dL
IgG4	54.7 mg/dL	11-121 mg/dL
Antinuclear antibody	Negative	Negative
T. Bil	5.9 mg/dL	0.4-1.2 mg/dL
D. Bil	1.0 mg/dL	0.2-1.2 mg/dL
BUN	43 mg/dL	8-20 mg/dL
Cre	1.11 mg/dL	0.65-1.07 mg/dL
Amy	604 U/L	44-132 U/L

TMA secondary to acute pancreatitis was considered most likely in view of the above findings, and additional biochemical analysis was performed to differentiate the causative disease (Table 2). The results revealed no decrease in the von Willebrand factor activity and the ADAMTS13 activity (a disintegrin and metalloproteinase with thrombospondin type 1 motif, member 13) and were negative for antiplatelet antibodies. Stool culture revealed no STEC. Therefore, it was determined that TMA was caused by aHUS, not STEC-HUS.

**Table 2:** Additional tests for differential diagnosis ADAMTS13: A disintegrin and metalloproteinase with thrombospondin type 1, motif 13.

Laboratory data		Reference range
von Willebrand factor activity	283%	60%-170%
Antiplatelet antibody	Negative	Negative
ADAMTS13 activity	63%	>10%
Urinary pneumococcal antigen	Negative	Negative

**Figure 5:**
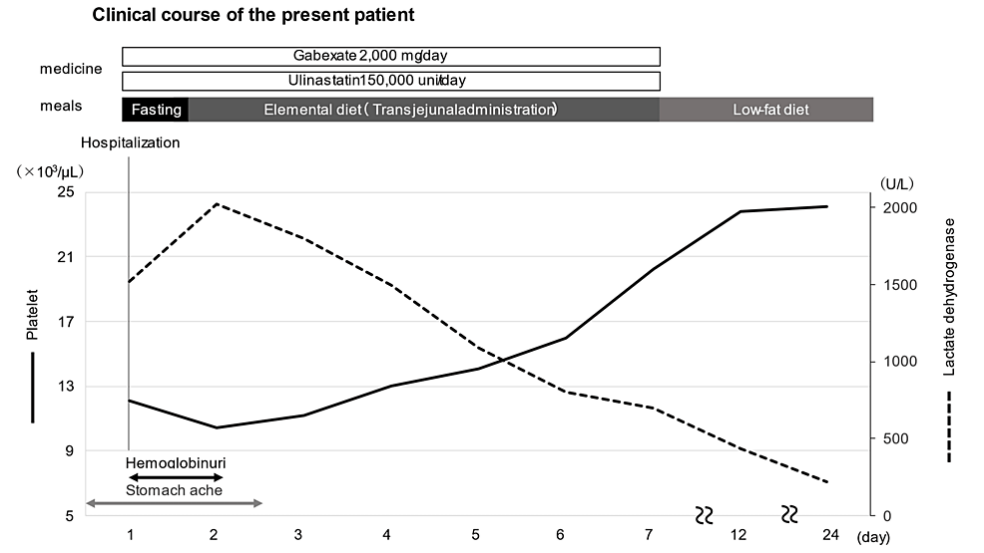
Clinical course of the present patient This illustrates the course of treatment, lactate dehydrogenase, and platelets. By the second day of illness, lactate dehydrogenase reached its peak and decreased over time, while platelets had an increasing trend over time.

The treatment course is shown in Figure 5. Treatment with ulinastatin 150,000 units/day, gabexate mesylate 2,000 mg/day, and meropenem 3 g/day were initiated on the day of the patient’s arrival. An ileus tube was placed in the jejunum. Follow-up with a urethral indwelling catheter for hemoglobinuria led to a trend toward improvement over time. The abdominal pain subsided from day 2 of hospitalization, and nutritional therapy was initiated with an elemental diet using an ileus tube. At this point in time, the hemoglobinuria had completely resolved, with a return to normal yellowish urine color. Pancreatic exocrine enzymes that were shown to be elevated in the biochemical analysis had improved over time, with no worsening of abdominal pain over the course of treatment. As parameters (such as bilirubin and LDH) indicative of hemolysis also steadily improved with the treatment for pancreatitis, no aHUS-specific therapy was provided.

Contrast-enhanced CT for reassessing the condition of pancreatitis on day 7 of hospitalization revealed that the inflammatory findings in and around the pancreas had already improved. Meropenem and ulinastatin were therefore discontinued on the same day, the ileus tube was removed, and the patient began oral ingestion of meals. As there was no subsequent recurrence of abdominal symptoms or worsening of biochemical results, the patient was transferred back to the initial hospital on day 26.

## Discussion

In reported cases of TMA preceded by acute pancreatitis, gallstones and heavy alcohol consumption were the most frequent triggers of acute pancreatitis [7], but a direct correlation between these triggers and TMA is not known, and pancreatitis itself may trigger TMA. Proposed mechanisms by which pancreatitis leads to TMA include the release of cytokines such as interleukin (IL)-1 or tumor necrosis factor-α and the influence of pancreatic exocrine protease-induced fibrinogen degeneration [8,9]. On the other hand, although cases are rarely reported and the possibility that TMA might induce pancreatitis cannot be completely ruled out, the fact that symptoms of pancreatitis are observed before TMA has been reported as evidence suggesting the possibility that secondary TMA is triggered by pancreatitis [10]. In the present case, the facts that hemoglobinuria (an indicator of TMA comorbidity) was not observed in the early stages when the abdominal pain had developed and that TMA gradually improved with only treatment for pancreatitis suggest that the TMA was triggered by pancreatitis.

There are various reports of autoimmune pancreatitis with autoimmune hemolytic anemia [11], TTP [12], HUS [13], and aHUS [6] in TMA secondary to pancreatitis, and drug-induced TMA [14] is also a possibility when drugs potentially causing TMA are used before or during hospitalization. However, TMA, including aHUS, must be assumed when pancreatitis is accompanied by elevated creatinine, hemolytic anemia, and low platelets, and peripheral blood smears or assay of ADAMTS13 levels, for example, should be employed for further differentiation of TMA. Pancreatitis with aHUS was diagnosed in the present case, and the aHUS also steadily improved with the treatment of pancreatitis. Supportive care is central to the treatment of pancreatitis with aHUS, but it has been reported that patients with aHUS as a complication of pancreatitis could be favorably treated with eculizumab, which is generally used to treat aHUS [15]. Moreover, inflammatory mediators such as IL-1, IL-6, and IL-8 were successfully managed with continuous renal replacement therapy [16], making these therapies potential treatment options for refractory aHUS.

## Conclusions

This is a report of an extremely rare case of an aHUS that appears to have been triggered by acute pancreatitis. Adults are rarely diagnosed with aHUS. Diagnosis is also further complicated when the progression of anemia or low platelet count is not very pronounced, but in the present case, the fact that intravascular hemolysis was noticed because of the hemoglobinuria provided a clue for the diagnosis. When hemolytic anemia is a complication of pancreatitis, the cause of hemolysis must be differentiated. When hemolytic anemia or thrombocytopenia of unknown etiology is observed, aHUS should be suspected, and clinicians should be aware that acute pancreatitis may be a potential cause of aHUS.
